# A randomized clinical trial of silver hydrofiber dressing versus collagenase ointment for venous ulcer: analysis of biofilm-producing bacteria and bacterial clonality^☆^

**DOI:** 10.1016/j.abd.2025.501162

**Published:** 2025-07-14

**Authors:** Taís Lopes Saranholi, Natalie Carlos Ferreira Mello Sampaio, Hélio Amante Miot, Stéfani Thais Alves Dantas, Vera Lucia Mores Rall, Luciana Patricia Fernandes Abbade

**Affiliations:** a Nursing Department, Faculty of Medicine, Universidade Estadual Paulista, Botucatu, SP, Brazil; b Faculty of Medicine, Universidade Estadual Paulista, Botucatu, SP, Brazil; c Department of Infectology, Dermatology, Imaging Diagnosis and Radiotherapy, Faculty of Medicine, Universidade Estadual Paulista, Botucatu, SP, Brazil; d Department of Chemical and Biological Sciences, Sector of Microbiology and Immunology, Instituto de Biociências, Universidade Estadual Paulista, Botucatu, SP, Brazil

**Keywords:** Clinical trial, Varicose ulcer, Wound infection

## Abstract

**Background:**

Superficial infection in venous ulcers (VU) hinders healing.

**Objective:**

To evaluate the action of hydrofiber dressing with silver (HAg) compared to collagenase ointment (Col) in VU.

**Methods:**

Randomized controlled clinical trial in which patients with VU with superficial infection were randomized to the intervention (HAg) or comparison (Col) group. After 30 days (T30), the primary outcomes evaluated were: rate of ulcers without signs of superficial infection, decrease in bacterial load, presence of biofilm-producing bacteria, and bacterial clonality.

**Results:**

Thirty-four patients (56 ulcers) were included ‒ 18 patients (28 ulcers) in the HAg group and 16 (28 ulcers) in the Col group. There was a reduction in ulcers with superficial infection in both groups over time but with no differences (*p* = 0.422). There was no decrease in total bacterial load over time (*p* = 0.054) or between the groups (*p* = 0.113). There was a reduction in the rate of ulcers with biofilm-forming bacteria over time (*p* = 0.047) but no differences between groups (*p* = 0.558). Regarding the clonality of *Staphylococcus aureus*, 92.8% of ulcers in the HAg group and 85% in the Col group, the clones identified at T0 were the same at T30 (*p* = 0.553). There was no change in the identity of *Pseudomonas aeruginosa* in any ulcer in either group.

**Study limitations:**

Short follow-up time.

**Conclusion:**

Both interventions improved the clinical and some microbiologic characteristics, but there was no difference between both interventions. In addition, most ulcers showed indistinguishable genetic profiles of *S. aureus* and *P. aeruginosa* between T0 and T30, with no difference between the groups.

## Introduction

Venous ulcers (VU) and other chronic ulcers are contaminated by microorganisms that may come from endogenous secretions, bandages, and their environment. Therefore, it is essential to understand the difference between colonization, critical colonization (superficial infection), and deep infection. The colonization will occur when bacteria replicate on the ulcer wound bed but do not cause damage to the host. However, the environment is susceptible to bacterial infections that happen when there is an increment in the microbial load on the surface of the ulcer (critical colonization or superficial/local infection). Consequently, the levels of pro-inflammatory cytokines increase and promote the deterioration of the granulation tissue and exudate in excess, hampering the healing process. Deep infection occurs when bacteria penetrate peri-wound tissues, replicate, and cause damage to the host, surpassing the immunological defense capacity, manifesting as erythema, edema, increase in temperature, pain at the perilesional region, and fever.^1^, ^2^, ^3^

Depending on the ulcer environment, different types of biofilms can be produced. Its formation starts when a bacterium in its planktonic form adheres to an exposed surface, which can be biotic or abiotic. After, the microorganisms connect among themselves and attach in an unreversible format. Then, they start to proliferate, activating the *quorum sensing*. The biofilm grows and differentiates itself, becoming a mature biofilm community, and presenting structural characteristics. Next, a matrix of water and polymeric substances such as polysaccharides, proteins, and extracellular DNA is formed. Finally, the mature biofilm starts a dispersion process with the detachment of cells or microcolonies. Dispersion can result in the colonization of new niches and, consequently, new sites of infections with biofilm.^4^

The resistance of biofilm-producing bacteria is a crucial problem in handling and treating chronic ulcers. In a mature biofilm, the bacteria grow slowly due to the deficiency of nutrients, resulting in resistance to topical and systemic antibiotics. Besides, penetration of these substances in the bacterial structure is reduced or incomplete and the biofilm in the ulcer is renewed quickly.^5^ Sharp debridement and silver dressing such as hydrofiber with silver (HAg) are lines of treatment described to combat biofilm.^6^ However, no method is entirely effective for biofilms, and scientific evidence is short to ensure the best form of removal from the chronic ulcer bed.

Products that promote enzymatic debridement, such as collagenase, are not described to combat biofilm. Collagenase is mentioned as an enzyme derived from the bacterium *Clostridium histolyticum*. It is effective and selective for acting in the collagen in the necrotic tissue without harming the granulation tissue. Thus, the use of it is pointed as possible after sharp debridement.^7^ Due to the frequent use of collagenase ointment in clinical practice as a routine treatment in health services, it is possible to use it as a comparative intervention. The study of VU's microenvironment and the search for interventions to combat superficial infection and biofilm are relevant aspects to consider when choosing the best therapeutic strategies to promote healing.

The objective of this study was to evaluate the action of the hydrofiber dressing with silver compared to collagenase ointment, both after sharp debridement, in the control of superficial infection, interfering in the formation of biofilm by *Staphylococcus aureus* and *Pseudomonas aeruginosa*, and in the clonality of the strains isolated in the initial and final time, in patients with VU that had used these interventions for 30-days. This time frame was chosen once the primary objectives were assessing the local superficial infection and microbiological outcomes instead of healing, which needs more time for follow-up.

## Materials and methods

A randomized, controlled, open-label clinical trial with a two-arm parallel group design, blinded outcome assessment was conducted with patients with VU with clinical signs of superficial infection. It was registered in the Brazilian clinical trials (ReBEC – RBR – 4kkq2h), and it was approved by the Research Ethics Committee of “The Medical School (FMB) of São Paulo State University (Unesp), Botucatu Campus,” under the number 3.055.504. Also, the study was reported following the CONSORT statement.^8^

It was performed on adult patients treated in the Chronic Ulcers Outpatient Clinic of Clinical Hospital of Botucatu Medical School ‒ Unesp, between October 2020 and November 2021.

### Participants of the study

The individuals who met all the following inclusion criteria and none of the exclusion criteria were eligible to be included. The criteria were: 1) Age equal or older than 18-years old; 2) Chronic VU (ulcer being active over six weeks) with clinical signs of superficial infection, following the mnemonic criteria rule NERDS^9^ if there were at least two of the following: i) N = Nonhealing wounds; ii) E = Exudative wounds; iii) R = Red and bleeding wound surface granulation tissue); iv) D = Debris-yellow or black necrotic tissue on the wound surface; v) S = Smell or unpleasant odor from the wound; 3) Agreement with the procedures proposed in the study; 4) Sign the written informed consent form (patient or guardian). Exclusion criteria: 1) Ulcers from other etiologies (peripheral arterial disease, hematologic, neoplastic, infectious causes, etc.); 2) Ulcers with deep tissue infections associated with erysipelas, cellulitis or lymphangitis due to the necessity of systematic antibiotic therapy; 3) History of hypersensitivity to proposed treatments; 4) VU associated to peripheral arterial disease that was classified when the Ankle-Brachial Index (ABI) was lower than 0.8 and absence of distal pulses, mainly tibial.

### Randomization and interventions

The randomization protocol was implemented using Software Rv 3.1.2, performed per patient, not per wound. To ensure allocation concealment, the randomization list was cared for by an unrelated individual in the clinical selection of participants. Therefore, the randomization was performed at basal Time (T0), and the participant could be allocated into the Collagenase group (Col group) or the Hydrofiber dressing with silver (HAg group).

After enrollment, the area of all ulcers was measured at baseline for all participants. Then, the ulcer was cleaned with a 0.9% saline solution. Next, the collection of material using a swab for microbiologic analysis and sharp debridement of the wound bed was done. This last procedure was performed with local topical anesthesia (lidocaine 4% cream) and kept under an occlusive dressing for 30-minutes. Afterward, the debridement was performed with a curette throughout the ulcerated bed.

Following the superficial debridement, the participants were randomized into two groups. The first group was made of participants treated with the hydrofiber dressing with silver (HAg ‒ AQUACEL® Ag + Extra™) in the shape of a pad with changes needed when saturation was reached (approximately four days). In the second group, the participants were treated with collagenase ointment (Col), which was not associated with the antibiotics (Kollagenase®). The presentation of collagenase was in ointment, and the quantity applied varied with the size of the wound. Still, it should be enough to cover all the wounded beds with a minimum layer of 2 mm, and after being covered with gauze, needing to be changed once a day.

In both groups, the compressive elastic band (Surepress®) was associated with the treatment, having to be applied once daily (in the morning and removed at the end of the day). The compressive elastic band is a compression system made of a unique layer, with pressure around 25 to 35 mmHg on the ankle. In addition, all the participants and the caretakers received instructions to take care of the wound at home during the study period.

### Outcomes

The outcomes evaluated after 30-days (T30) were the following.

Primary: (i) Rate of wounds with no sign of superficial infection following the clinical criteria in the mnemonic rule NERDS;^9^ (ii) Microbiologic analysis – the rate of ulcers with reduction of bacterial load; rate of ulcers with biofilm-forming bacteria; rate of ulcers with bacterial identity changes (clonality).

Secondary: (i) Clinical improvement of VU assessed by the PUSH scale (Pressure Ulcer Scale for Healing).^10^ The total score can range from 0 to 17; higher scores represent the worst conditions for the ulcer; (ii) Decrement of ulcer area using the planimetric method; (iii) Healing rate assessed clinically by the observation of the total epithelialization without the presence of crusts at the place of VU; (iv) Adverse local events related to the interventions.

All the outcomes were evaluated by an independent clinician blinded to treatment allocation.

### Microbiologic analysis

The ulcers were irrigated with a 0.9% saline solution over mechanical pressure to collect the samples. A sterile gauze mold with a central opening of 1 cm^2^ was placed onto the wound bed with the appearance of the most unviable tissues. The swab was moistened with a sterile saline solution (0.9%) and rubbed along the exposed area. Later, the swab was conditioned in a tube with 3 mL of sterile 0.9% saline solution, which was placed under refrigeration and immediately transferred to microbiologic analysis.

The microbiologic analyses were made in the Laboratory of Microbiology of the Botucatu Institute of Biosciences (São Paulo State University ‒ Unesp). In this study, the most prevalent biofilm-forming bacteria in the VU mentioned in the literature ‒ *S. aureus* and *P. aeruginosa ‒* were selected to be analyzed. The *Staphylococcus epidermidis* was also assessed for being a bacterium that forms biofilm.^11^ The other bacteria in the cultures were only described as present and their load. Still, they were not evaluated regarding the ability to form biofilm and the bacterial identity between T0 and T30. To calculate the number of colony-forming units per square centimeter (CFU/cm^2^), the number of confirmed colonies was multiplied by 10 (100 μL inoculum) and then by 3 (total of 3 mL in the swab tube).

#### Biofilm production on polystyrene microplate

For each culture of *S. aureus*, *S. epidermidis*, and *P. aeruginosa*, growth in BHI under 35 °C/24 hours was diluted to approximately 1.5 × 10^8^ CFU (0.5 MacFarland scale), with the support of Densicheck (Biomeriéux For), using the BHI supplementing with 0.5% of glucose. The Optical Density (OD) was measured using an ELISA reader (Babsystems, MultiSkan EX) at 570 nm. BHBI not inoculated was used as a negative control of the reactions. The positive control of *P. aeruginosa* was the ATCC 9027 and the Staphylococcus was *S. epidermidis* ATVV 35983. From the average of the four repetitions (OD) and according to the relation between the OD (test sample) and the OD (negative control), the strains were classified into the following categories: no biofilm producers, weak, moderate, or strong biofilm producers, as mentioned by Stepanovic et al.^12^

#### Identification of the bacterial typing by Pulsed Field Gel de Eletroforese (PFGE)

The molecular typing using the PFGE technique was performed for the *S. aureus and P. aeruginosa bacteria* when the same species were found in one ulcer on T0 and T30. For the strains of *S. aureus,* the methodology used was described for PulseNet.^13^ For the strains of *P. aeruginosa*, the methodology of Gram-negative of Selim et al.^14^ was employed.

### Sample size and statistical analysis

For the sample size, the authors assumed that 20% of VU would remain with superficial infection in the HAg group and 60% in the Col group. The difference between the groups was based on previous studies that found almost 80% of superficial infection was controlled with HAg dressing^15^, ^16^ In the Col group, the authors assumed only 40% control of the infection since its action is enzymatic debridement.^7^ Considering 80% of statistical power and a two-tailed significance level of 95%, 27 VU by each intervention group was necessary.

The statistical analysis was by intention to treat. It was executed respecting the assumptions determined by the variables' results, characteristics, and behavior. The binomial variables were compared by Chi-Square (c^2^) and Fisher’s Exact tests. The Student's *t*-test or Mann-Whitney *U* test compared the numerical variables. The normality was assessed by the Shapiro-Wilk test. The Longitudinal follow-up of ulcer areas, the PUSH score, was reached between the groups by a generalized linear mixed model with an adequate probability distribution for each variable. The magnitude of the effect on nominal data was assessed by the Relative Risk (RR) and its Confidence Interval of 95% (95% CI).^17^ Data were analyzed using the software SPSS 22.0 and considered a significant value of p < 0.05.

## Results

**Fig. 1:**
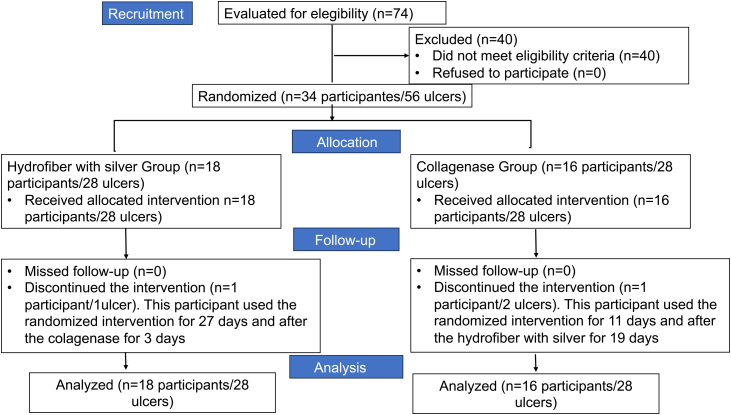
Flowchart of the study according to CONSORT.

During the study period, 74 participants were evaluated to determine if they met the eligibility criteria (Fig. 1). Among them, 40 were excluded for not meeting the eligibility criteria. Thus, 34 participants with 56 venous ulcers were eligible and randomized for the study. Eighteen participants with 28 VU were randomized for the HAg group and 16 participants with 28 VU for the Col group. After 30 days of follow-up, one participant from the HAg group with one ulcer and one from the Col group discontinued the interventions.

Table 1 presents the demographic and clinical data from the participants in the basal time (T0), distributed by group of study. It is observed that the study groups are homogenous and not statistically different among the variables.

**Table 1:** Distribution of the 34 participants (56 ulcers) by study group according to demographic and clinical data on basal Time (T0).

Variables related to patients	Group		Total, n = 34 (%)	p-value
	Col, n = 16 (%)	HAg, n = 18 (%)		
Average age (in years) (±SD)	70.7 (±9.2)	68.6 (±8.9)	69.6 (±9.0)	0.94
Women	11 (68.8)	13 (72.2)	24 (70.6)	0.83
Men	5 (31.3)	5 (27.8)	10 (29.4)	0.83
SAH	11 (68.8)	14 (77.8)	25 (73.5)	0.58
DM	5 (31.3)	6 (33.3)	11 (32.3)	0.90
Vascular surgical procedure history	6 (37.5)	4 (22.2)	10 (24.49)	0.36
Reduced ankle mobility	9 (56.2)	10 (55.5)	19 (55.8)	0.97
History of DVT	5 (31.3)	3 (16.7)	8 (23.5)	0.35
ABI - median (max‒min)	1.07 (0.80–1.50)	1.05 (0.93–1.21)	1.06 (0.80–1.50)	0.25

Variables related to the wounds Median (min–max)	n = 28 ulcers	n = 28 ulcers	Total (n = 56 ulcers)	p-value
Ulcers’ time (months)	24.0 (1.0–168.0)	42.0 (2.0–600.0)	30 (1.0–600.0)	0.25
Ulcer’s area (cm^2^)	10.3 (1.0–96.9)	14.3 (1.0–123.3)	11.2 (1.0–123.3)	0.74

SHA, Systemic Arterial Hypertension; DM, Diabetes Mellitus; DVT, Deep Venous Thrombosis; ABI, Ankle Brachial Index.

### Primary outcomes

#### Rate of ulcer without clinical signs of superficial infection at T30

There was a reduction of ulcers with criteria of superficial infection in both groups over time. In 29/56 ulcers (51.8%; 95% CI 39.0%‒64.3%), there were no signs of superficial infection at T30, 13/28 from HAg group (46.4%; 95% CI 28.6%‒65.4%) and 16/28 from Col group (67.1%; 95% CI 40.0%‒73.4%), not having statistic difference between the groups (p = 0.422). The RR for non-superficial infection at the end of 30 days among the HAg group compared to the Col group was 0.81 (95% CI 0.39–1.68).

#### Rate of ulcers with bacterial load reduction

**Fig. 2:**
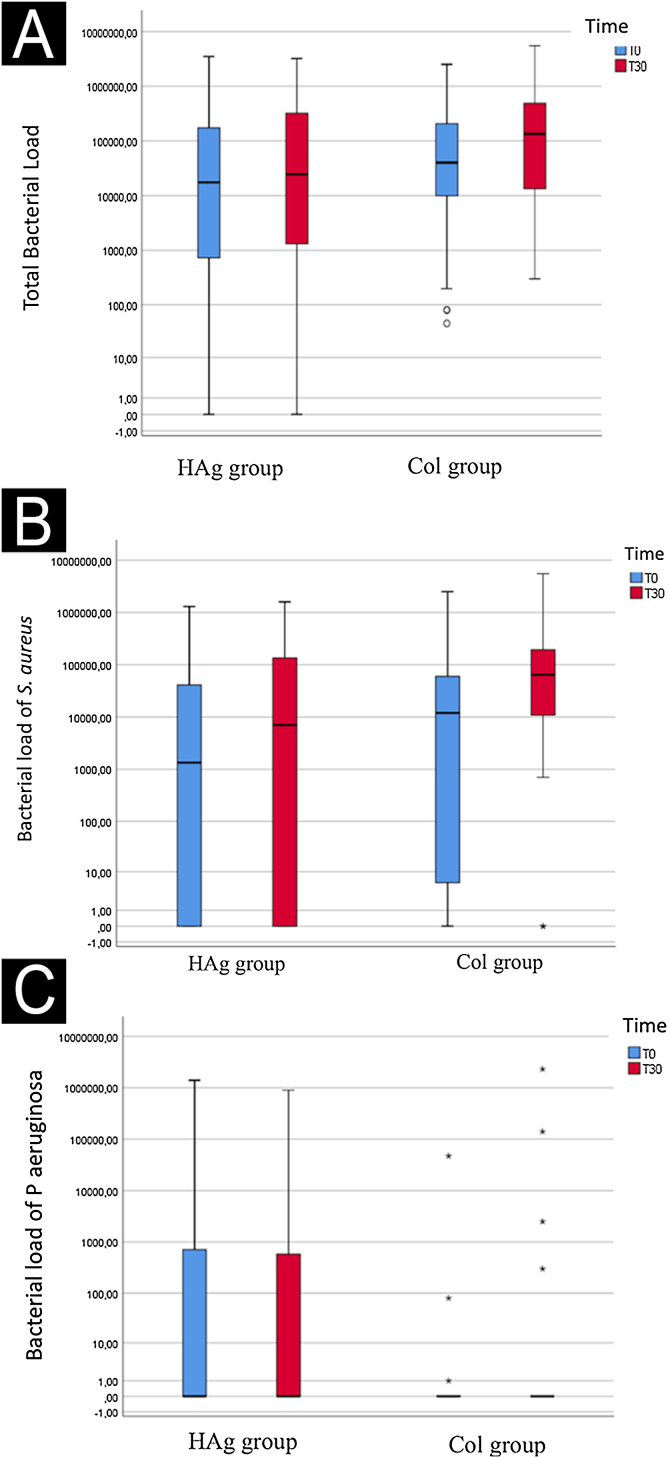
Log of the bacterial load (CFU/cm^2^) of the total evaluated species (A), *S aureus* (B), and *P. aeruginosa* (C) at baseline (T0) and after 30-days (T30), in the ulcers of the Hydrofiber with silver (HAg) and Collagenase (Col) group. The bacterial load over time (mean before vs. mean after) had a marginal significance (p = 0.054); however, it did not differ between the groups (p = 0.113).

It was possible to identify that no decrease of the bacterial load over time occurred (p = 0.054) nor did it happen between the groups (p = 0.113) (Figs. 2 A, B, and C).

At T0, 37/56 of ulcers had growth of *S. aureus* (66.1%, 95% CI 53%‒77.1%); in the HAg group occurred in 16/28 (57.1%; 95% CI 39.1–73.5%) and the Col group in 21/28 ulcers (75%; 95% CI 56.6%‒87.3%). At T30, 46/56 had growth of *S. aureus* (82.1%; 95% CI 70.1%‒90%), in the HAg group occurred in 20/28 ulcers (71.4%; 95% CI 52.4%‒84.75%) and the Col group in 26/28 ulcers (92.8%; 95% CI 77.5%‒98.0%). There was no difference with statistical significance over time (p = 0.345) and according to the intervention groups (p = 0.336).

As for *P. aeruginosa*, on T0, there was a growth in 16/56 ulcers (28.5%; 95% CI 18.4%‒41.5%); in the HAg group, it occurred in 11/28 (39.3%; 95% CI 23.6%‒57.6%) and the Col group occurred in 5/28 (17.8%; 95% CI 7.9%‒35.6%). At T30, 13/56 ulcers had growth of *P. aeruginosa* (23.2%; 95% CI 14.1%‒35.8%), in the HAg group occurred in 7/28 ulcers (25%; 95% CI 12.7%‒43.3%) and the Col group in 6/28 ulcers (21.4%, 95% CI 10.2%‒39.5%). The *S. epidermidis* only occurred in three ulcers on T0.

#### Rate of ulcers with biofilm-forming bacteria

There was a reduction in the rate of ulcers with biofilm-forming bacteria from T0 to T30 (p = 0.047), but there was no statistically significant difference between the groups at T30 (p = 0.558).

**Fig. 3:**
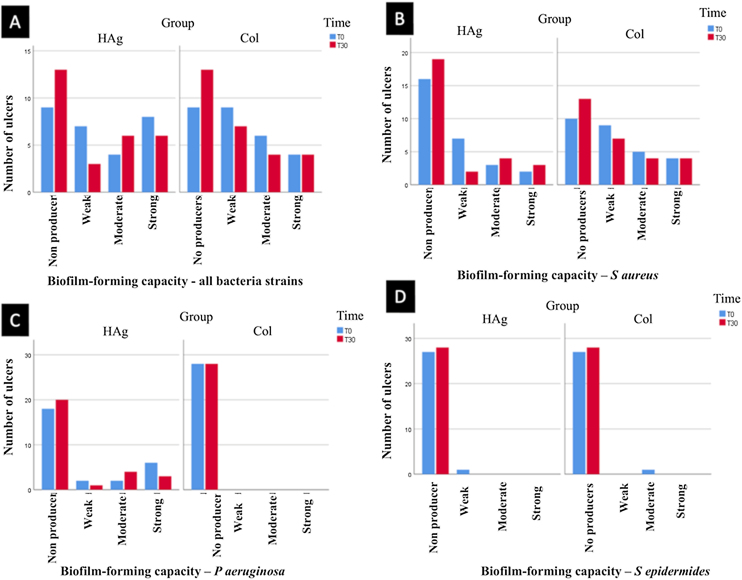
Classification of bacteria strains that form biofilm. (A) Classification of all bacteria strains evaluated together (*S. aureus*, *P. aeruginosa*, and *S. epidermidis*). (B) The capacity of the *S aureus* to form biofilm. (C) The capacity of the bacteria *P. aeruginosa* to form biofilm. (D) The capacity of the bacteria *S epidermidis* to form biofilm. There was a reduction in the rate of ulcers with biofilm-forming bacteria from T0 to T30 (p = 0.047), but there was no statistically significant difference between the groups at T30 (p = 0.558).

At T30, in the HAg group, 13/28 (46.4%; 95% CI 28%‒63%) did not present bacteria that form biofilm; in the Col group also 13/28 (46.4%; 95% CI 28%‒63%). Thus, the RR of ulcers with bacteria that do not form biofilm was 1.0 (95% CI 0.46–2.16; p = 1.000). The results of the biofilm production by a strain of bacteria are shown in Fig. 3.

#### Rate of ulcers with change in the bacterial identity

The molecular typing using the PFGE technique was performed for the bacteria (*S. aureus* and *P. aeruginosa*) from the same species in a specific ulcer on T0 and T30. However, typing was not performed for S epidermidis since there was no growth of this bacterium on T0 and T30 in the same ulcer.

**Fig. 4:**
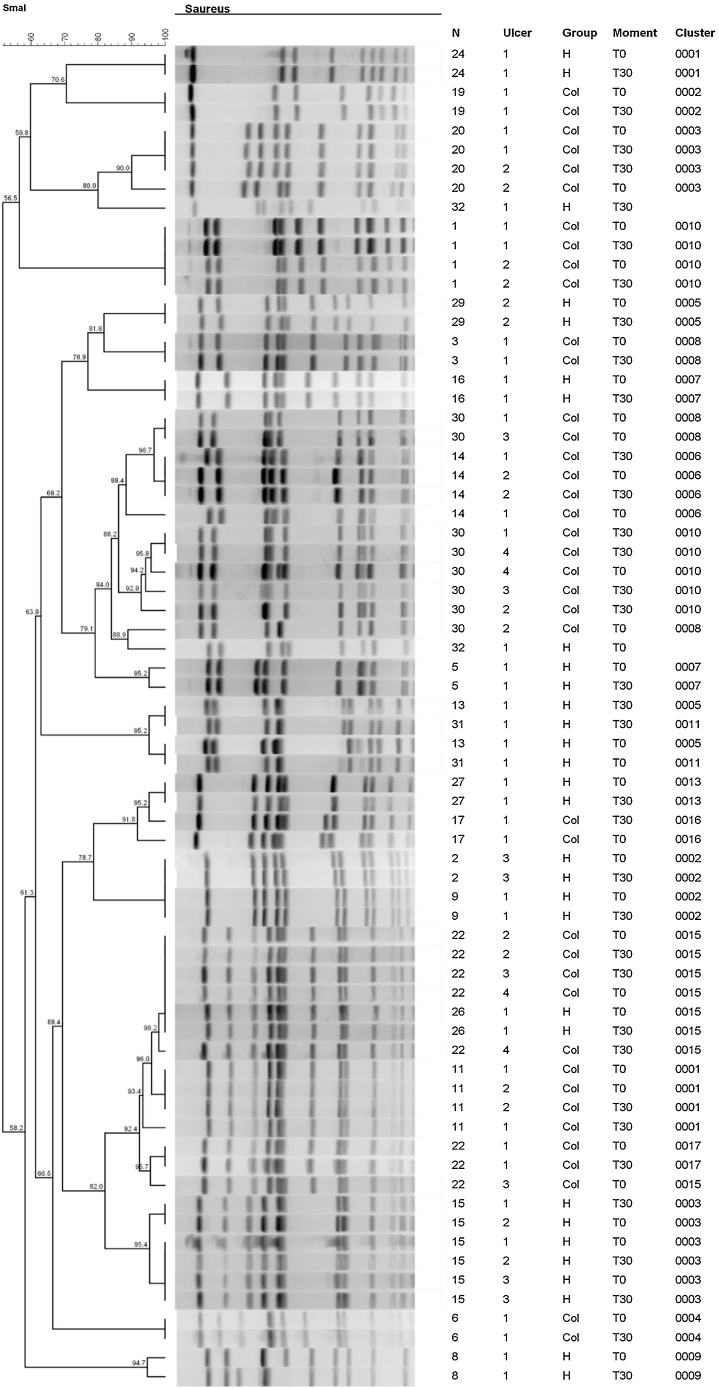
Dendrogram of genetic similarity of ulcers with *S aureus.* The RR to have *S. aureus* with a change in the identity is 0.58 (95% CI 0.10–3.31; p = 0.553).

Fig. 4 shows the results of the dendrogram of genetic similarity of *S. aureus* of ulcers that had growth of this bacterium on T0 and T30. It was possible to identify the existence of 17 total clones of bacteria in 22 participants at different moments and groups of this study. In that regard, there was a significant similarity, over 80%, in most ulcers, so the identical clones were identified on T0 and at T30. The clones of *S. aureus* identified on T0 were the same identified at T30 in 13/14 ulcers (92.8%; 95% CI 68.5%‒98.7%) in the HAg group and in 17/20 ulcers (85%; 95% CI 64.0%‒94.8%) in the Col group. The RR to have *S. aureus* with a change in the identity is 0.58 (95% CI 0.10–3.31; p = 0.553); therefore, there is no difference with statistical significance between the groups.

**Fig. 5:**
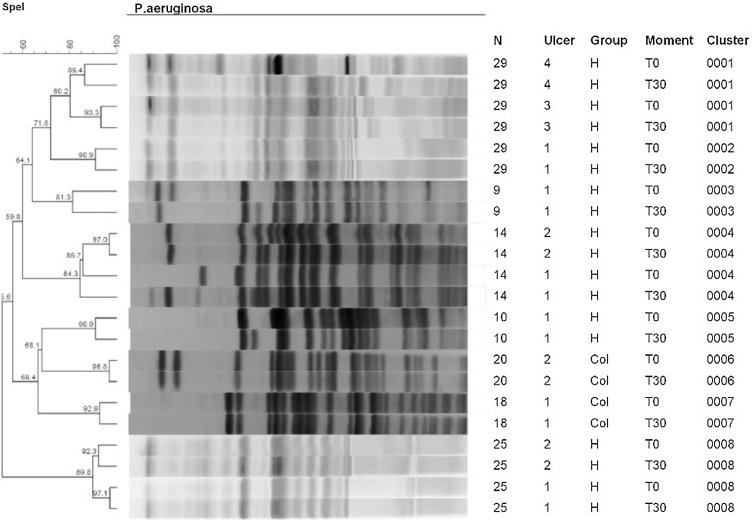
Dendrogram of genetic similarity of ulcers with *P. aeruginosa.* There was no change in the identity of *P. aeruginosa* in any ulcer in both groups.

The PFGE analyses on strains of *P. aeruginosa* are observed in the dendrogram of Fig. 5. This dendrogram was created using one final partition of 8 groupings with a level of similarity of over 80%. All wounds of the dendrogram identified a high similarity of the clones on T0 and at T30. It was possible to identify eight clones in 7 participants with 11 ulcers. Of these eleven ulcers, 9/28 were from the HAg group (32.1%) and 2/28 (7.14%) from the Col group. There was no change in the identity of *P. aeruginosa* in any ulcer in both groups.

### Secondary outcomes

#### Clinical improvement of VU

In the HAg group, the average PUSH value on T0 was 13.5 (±2.5), at T30, it was 12.7 (±3.3). In the Col group, the average value on T0 was 13.5 (±2.5), and at T30, it was 12.4(±2.9). Both groups showed improvement in the score over time (p = 0.001); however, there was no statistically significant difference between the groups (p = 0.0668). The average percentile score reductions of PUSH in the HAg group was -6.8 (95% CI −1.3 to −13.0); in the Col group, it was −7.6 (95% CI −1.10 to −14.1), confirming that there was no difference between the groups.

#### Reduction of ulcer area and total healing

The average reduction in ulcer area was 0.78 cm^2^ (95% CI -0.20 to -1.36) in the HAg group, and 1.1 cm^2^ (95% CI −0.20 to −2.00) in the Col group. There was a reduction in the area for both groups over time (p = 0.001) but no difference in statistical significance between the groups (p = 0.786). There were no ulcers with total healing in both analyzed groups.

### Adverse events

There were no adverse events related to both interventions. One patient with four ulcers in the Col group had secondary myiasis, but this event was unrelated to the intervention.

## Discussion

Evaluating interventions that act in the bacterial load and biofilms of VU is crucial, for there is a high prevalence of 62% to 89% of biofilms in chronic wounds.^18^ This randomized clinical trial was specifically designed to evaluate, in a short period, the efficacy of the Hydrofiber dressing with silver on clinical outcomes such as local infection and microbiologic such as reduction of the bacterial load and inhibition of bacteria biofilm producers. It was assessed if the dressings could eliminate the bacteria that were initially colonizing the ulcer, using the bacterial clonality evaluation, comparing basal outcomes with the follow-up's final time.

The main results show that both interventions, Hydrofiber dressing with silver and collagenase ointment, have promoted the reduction of ulcers with criteria of superficial infection, a decrease in ulcer rate with biofilm-forming bacteria over time, but with no statistically significant difference between the groups. On the other hand, there was no reduction in total bacterial load over time, but there was a marginal significance (p = 0.054). So, the lack of detection of a difference may have been due to the small sample (underpowered). However, there was no suggestion of a difference between the groups.

Most of the ulcers presented indistinguishable PFGE profiles between T0 and T30, showing a lack of action to eliminate the bacteria that were initially colonizing the ulcer. The initial hypothesis presumed that the Hydrofiber dressing in silver would be superior to the Collagenase ointment since there is more evidence of its action against superficial infection and biofilms due to its antimicrobial properties.^15^, ^16^ Dressings containing silver are known for reducing the bacterial load and acting as an antimicrobial barrier to avoid higher contamination of the wound by external microorganisms. These dressings have a lower risk of bacterial resistance when there is an adequate dose of silver.^15^ In addition, silver acts against Gram-negative bacteria, Gram-positive bacteria, fungi, and viruses,^19^ even though there have been recent reports of bacterial resistance to silver.^20^

The clinical trials with dressings with silver generally evaluate outcomes of healing. However, the results related to the local control of the infection and microbiologic effects still need to be assessed, which hampers the present findings' comparison.

A Cochrane systematic review with meta-analyzes from 2010 concluded that there was no evidence to confirm if products with silver can promote wound healing.^21^ However, other systematic revisions from recent years have found different results. A Cochrane systematic review in 2018 about dressings and topical agents for the treatment of VU found evidence that the dressings with silver can increase the probability of VU healing compared to non-adherent dressings (RR = 2.43; 95% CI 1.58 to 3.74) with evidence of moderate certainty.^22^ The systematic review made in 2020 about dressings with silver, specifically for VU, included eight randomized clinical trials, totaling 1,057 patients, 526 participants with dressings with silver, and 531 controls. The evaluated outcomes were clinical, such as wound area reduction and healing. There was evidence that dressings with silver can increase the healing rate of VU and improve its recovery in a short period. However, compared to other dressings, clinical trials with a long time of follow-up are necessary to confirm if the dressings with silver are superior to the complete healing of the wound.^23^

In the present study, both groups were submitted to debridement by superficial surgical curettage before the randomization, which can explain the similar results between both groups. Debridement represents a treatment strategy against biofilms. Although, it cannot be eradicated because its formation occurs a few hours after the procedure. Thus, it is not recommended the isolated use. Still, it is a strategy that supports the best efficacy of topical therapies against biofilm performed after debridement, as it opens a window of opportunity.^24^

Biofilm and bacterial load of ulcers promote a delay in healing and can cause de development of infections.^25^ Therefore, the knowledge of the most frequent microorganisms in chronic ulcers shows a way to create wound treatment strategies. The clinical-laboratory correlation regarding the bacterial flora of chronic ulcers is a challenge. The microbiologic sample can be analyzed in a qualitative and quantitative approach. In this study, the swab was performed in a predetermined area, making the quantitative analyses and identification of bacteria species possible. The swab technique is less invasive and can be used for qualitative, semi-quantitative, or quantitative analyses. It is mainly employed in outpatient settings.^26^

The swab technique is less invasive and can be used for qualitative, semi-quantitative, or quantitative analyses. It is predominantly employed in outpatient settings.

The most frequent bacteria in chronic ulcers are *S. aureus* and *P. aeruginosa.* At the same time, the prevalence of other types varies in the different studies.^27^ The ulcer can evolve into an infection of deeper soft tissues when the bacteria reach a critical level of 10^5^ unities creators of colony per gram of tissue. When the number of bacteria increases over this level, the probability of infection increases as the host's immune system cannot control the bacteria's proliferation anymore.^28^ The study that analyzed the microbiological characteristic of 754 VU, using the swab in a 1 cm^2^ area, in a sample of 636 (84.3%) patients presented ulcers with positive for culture.^29^ Among the pathogens identified in the study, *P. aeruginosa* was detected in 28.6% of cultures. In the present study, about 66% of the ulcers were colonized by *S. aureus* and 28.5% by *P. aeruginosa.*

The molecular typing, by the PFGE technique performed in this study, demonstrated the high similarity of clones on T0 and T30 in the strains of *S. aureus* and *P. aeruginosa.* Only one ulcer in the Hydrofiber dressing with a silver group and three from the Collagenase group presented different genetic profiles of *S. aureus*. PFGE profiles of *P. aeruginosa* were indistinguishable in any ulcers from both groups. Then, the present results show that the dressings are not efficacy in changing the bacterial clonality. Another study evaluated the colonization in wounds by *P. aeruginosa* via PFGE; it identified the bacteria in 48 isolated cultures of chronic ulcers that performed different types of dressings, including silver, demonstrating multi-resistance in three of them. *P. aeruginosa* was present in 75% of the samples of chronic wounds. In most ulcers, the strains showed the same genetic characteristics on both analyzed moments, indicating that the wound beds remained colonized and that the clones persisted for more than a month of treatment, a similar result found in this study. Most bacteria had virulence genes associated with the high potential to establish infection.^30^

The present study has some limitations. First, the follow-up time of 30-days is considered insufficient to evaluate the evolution of the healing process of chronic ulcers. However, the primary outcomes were associated with the assessment of the local superficial infection and microbiological outcomes, which could be achieved in the proposed period. Another limitation is the material collection procedure for the microbiological analyses, as the gold standard is tissue biopsy due to the capacity to evaluate the deeper bacteria. However, the biopsy has the disadvantage of being more invasive, painful, and expensive than the swab technique.^11^ Also, the swab was collected in a small area of the wound bed. The distribution of bacteria and biofilms in chronic wounds may not be even. Therefore, one unique biopsy sample or swab in a small area can influence the results because other regions of the same injury can show different results. However, the swab was performed in the wound bed with traces of unviable tissue, increasing the chances of representing the site with the highest bacterial load and biofilm.

The results of this study allow for discussing the efficacy of interventions referred to as strategies for treating ulcers with superficial infection and biofilm, with clinical and microbiological evaluation of essential outcomes related to wound infections.

## Conclusion

Both interventions improved the clinical and some microbiologic characteristics over time, but there was no difference between both interventions after 30-days of follow-up. In addition, most ulcers showed indistinguishable genetic profiles of *S. aureus* and *P. aeruginosa* between T0 and T30, with no difference between the groups.

## ORCID ID

Taís Lopes Saranholi: 0000-0002-2397-0646

## Authors’ contributions

Taís Lopes Saranholi: Study design and planning; data collection, analysis, and interpretation of data; writing of the article; critical review of the literature; critical review of the manuscript; approval of the final version.

Natalie Carlos Ferreira Mello Sampaio: Data collection, analysis, and interpretation of data; writing of the article; critical review of the literature; critical review of the manuscript; approval of the final version.

Hélio Amante Miot: Analysis and interpretation of data; Statistical analysis; writing of the article; critical review of the manuscript; approval of the final version.

Stéfani Thais Alves Dantas: Data collection, analysis, and interpretation of data; writing of the article; critical review of the literature; critical review of the manuscript; approval of the final version.

Vera Lucia Mores Rall: Data collection, analysis, and interpretation of data; writing of the article; critical review of the literature; critical review of the manuscript; approval of the final version.

Luciana Patricia Fernandes Abbade: Study design and planning; data collection, analysis, and interpretation of data; writing of the article; critical review of the literature; critical review of the manuscript; approval of the final version.

## Financial support

This study was financed by the Research Support Foundation of the State of São Paulo (FAPESP). Brazil. The funding agency had no role in the study design; in the collection, analysis, and interpretation of data; in the writing of the report; and in the decision to submit the article for publication.

## Conflicts of interest

None declared.
